# Applicability of academic real-world data research in the case studies of the HTx project to practical health technology assessment work

**DOI:** 10.3389/fphar.2026.1650552

**Published:** 2026-03-13

**Authors:** Carl Björvang, Johan Pontén, Anders Viberg, Andrea Manca, Georgia Salanti, Pekka Siirtola, Gema García-Sáez, Saskia Knies, Diana Delnoij, Dalia Dawoud, Jamie Elvidge, Lifang Liu, Bertalan Németh, Zoltán Kaló

**Affiliations:** 1 Dental and Pharmaceutical Benefits Agency, Stockholm, Sweden; 2 Centre for Health Economics, University of York, York, United Kingdom; 3 Institute of Social and Preventive Medicine, University of Bern, Bern, Switzerland; 4 Biomimetics and Intelligent Systems Group, University of Oulu, Oulu, Finland; 5 Bioengineering and Telemedicine Group, Centrode Tecnología Biomédica, ETSI de Telecomunicación, Universidad Politécnica de Madrid, Madrid, Spain; 6 National Healthcare Institute, Diemen, Netherlands; 7 Erasmus School of Health Policy & Management, Erasmus University Rotterdam, Rotterdam, Netherlands; 8 Faculty of Pharmacy, University of Cairo, Cairo, Egypt; 9 Science Policy and Research Programme, National Institute for Health and Care Excellence (NICE), London, United Kingdom; 10 Department of Statistics, European Organisation for Research and Treatment of Cancer, Brussels, Belgium; 11 Syreon Research Institute, Budapest, Hungary; 12 Center for Health Technology Assessment, Semmelweis University, Budapest, Hungary; 13 Center for Pharmacology and Drug Research & Development, Semmelweis University, Budapest, Hungary

**Keywords:** cost-effectiveness, health technology assessment, HTx, randomized controlled trial data, real-world data, transferability, treatment effect

## Abstract

**Introduction:**

Next-Generation HTA (HTx) is a recently finalised EU project which aimed to connect academic HTA researchers with HTA practitioners.

**Methods:**

This paper studies the applicability of the academic RWD research conducted through four case studies within the HTx project to practical HTA work. As a first step HTA Agency representatives of the HTx consortium sent a survey out to each case study leader, enquiring about the usefulness of their RWD research methods for HTA agencies in various situations, and barriers to their utilisation. The second step was to conduct follow-up interviews with the case study leaders, to further understand the new RWD research methods and their applicability to HTA practices.

**Results:**

The results show a great variety in when and how the methods could be used for practical HTA evaluations. Generally, there was a high potential for the RWD research methods to be used to estimate treatment effects and cost-effectiveness, while they were less adapted for estimating natural disease progression and identifying relevant comparators. However, there were significant barriers to the use of these methods for practical HTA evaluations, even in the aspects of evaluations the methods were designed to handle. These barriers ranged from the availability of RWD and need for partial reliance on RCT data, to required expertise in areas such as data evaluation, statistics and medical knowledge.

**Discussion:**

The case studies show that RWD can be used for a range of HTA aspects and in various situations. However, no RWD research method is a silver bullet that is applicable for all aspects in all situations. As such, they can make significant contributions to the work of HTA agencies, but as part of a wider tool set.

## 1 Introduction

With aging populations, advanced therapy medicinal products and new treatments for highly prevalent diseases and conditions such as obesity and Alzheimer’s, healthcare systems face substantial financial pressures in the coming years. There is also a trend that less comparative evidence is available for the appraisal of new pharmaceuticals to health technology assessment (HTA) agencies, (Chauca et al., 2023), partly because the number medicines which are registered based on single arm trials is increasing over time (Patel et al., 2021). As such, it is imperative for healthcare payers to find ways to optimize their spending. Utilisation of real-world data (RWD) could be a vital tool, as it can improve the assessment of benefits/added value of a given treatment in the general population. However, methods to utilize RWD in HTA, which are often developed in academic settings, need to be applicable in the practice of organisations and agencies conducting practical HTA work. One of the aims of the recently completed HTx (Next-Generation HTA) EU funded Horizon 2020 project (see https://www.htx-h2020.eu/) was to connect academic HTA researchers with HTA practitioners. This study was conducted to understand how applicable the academic RWD research conducted within the HTx project is to HTA practices.

There are various definitions of RWD (Makady et al., 2017a). In the context of this paper, we define RWD as data that is gathered on patients being provided a given treatment in the course of regular healthcare settings. Without RWD, HTA decision makers are often reliant on data from randomised controlled trials (RCTs), settings specifically designed to evaluate the efficacy and safety of that particular treatment often performed among selected patient population prior to HTA evaluation. RCT data has a number of advantages over RWD. For example, an RCT has high internal validity, as each individual is randomized to a treatment which results in causality between treatment and evaluation of efficacy and safety. Within an RCT patient follow-up is often monitored more closely, resulting in fewer missing values and other data abnormalities.

However, RWD also has a number of advantages over RCT data. RWD has the potential for higher external validity, since it is gathered in regular healthcare settings, therefore, it is better at reflecting actual treatment provision and outcomes. RWD can also be better at following individuals over longer stretches of time, increasing the likelihood of information on subsequent treatments and time-varying patient characteristics. The quantity of treatments, and their diversity in terms of patient populations and healthcare settings, can also be far greater than what would be economically feasible for RCTs.

For HTA agencies, the wider the adoption of the treatment in other countries provides an advantage to consider more data in the initial assessment, if the evidence generated from RWD is transferable across countries. In general, the use of RWD could improve HTA assessments for that treatment over time. Hence, while RWD is not a replacement for RCT data, especially not for the countries in which a treatment is first introduced, it can be an important compliment to RCT data in understanding the effectiveness of a treatment within regular healthcare settings.

RWD are observational data obtained from routine clinical practice, while (RWE) entails evidence obtained from the analysis of RWD (Németh el al. 2023). The source of the data, real-world or RCT, determines the analytical approach, and so this paper focuses on the applicability of academic RWD-based research methods to HTA practices.

**FIGURE 1:**
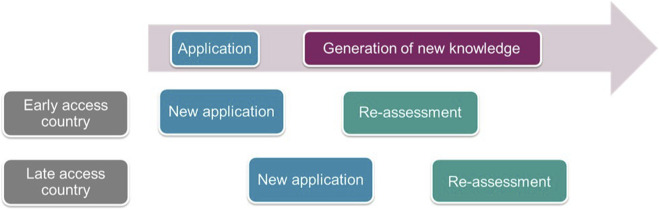
Model of reimbursement applications and knowledge generation in early and late adopter countries of new health technologies over time.

It is important to note that, since HTx was designed as a collaboration between academic HTA researchers and HTA practitioners, it ought to represent a best-case scenario for applicability. As such, it is unlikely that results from other academic research methods will be as easily applicable in HTA practices. Note also that this paper is not aimed to evaluate results of the individual HTx case studies in a specific jurisdiction, but considered the applicability of RWD research methods in a broader context. Local RWD is typically not available before the widespread use of a new technology, e.g., in case of new reimbursement applications, but can be available in case of HTA re-assessment. On the other hand, international RWD may be utilized in countries with delayed access to the technology even during the initial HTA assessment. As such, the applicability of RWD research methods depends on whether the HTA covers a new application or a re-assessment and whether the HTA is conducted in an early or late access country (see Figure 1).

Applicability, in the context of this paper, refers to the ability of HTA agencies to utilize an RWD research method developed in an academic setting in their HTA practices. Transferability refers to the ability to transfer RWD research from one country to another, either as raw data or as processed RWE. As such transferability can be a key part of applicability, as it means the HTA practitioner can rely on RWD research from other jurisdictions. However, other aspects of RWD research methods, both strengths and weaknesses, influences whether the HTA practitioner finds a given method useful. As such, transferability is strictly neither sufficient nor necessary for applicability of RWD research methods to HTA practices.

## 2 Materials and methods

This study was undertaken using a mixed methods research approach to understand the applicability of research methods in the HTx case studies to HTA practices. As a first step, HTA agency representatives of the HTx consortium sent a questionnaire with open and closed questions to each case study leader. As a second step, semi-structured follow-up interviews were carried out with case study leaders to elaborate on their responses. Findings from both the questionnaire and interviews were triangulated to allow the study team to both obtain comparable information across the case studies and to gain further insights about each individual case study.

### 2.1 About HTx

HTx was a Horizon 2020 project with the main aim to “create a framework for the Next-Generation HTA to support patient-centred, societally oriented, real-time decision-making on access to and reimbursement for health technologies throughout Europe” (HTx, 2020). It featured four case studies with special focus on the RWD usage in the technology appraisal of different technologies described in Table 1.

**TABLE 1:** Overview of HTx case studies, including disease areas, treatments, data sources, research methods and key outputs.

Case study and lead partner	Disease area	Treatment	Data sources	Research methods	Output
1 European Organisation for Research and Treatment of Cancer (EORTC)	Head and Neck Cancer	Proton therapy	Cancer registry data from the Netherlands, Ireland, Spain, and Slovenia	Comparative studies of a selection of normal tissue complication probability (NTCP) models	NTCP model
2 Universidad Politécnica de Madrid	Diabetes	Heterogeneous Care	The Clinical Practice Research Datalink (CPRD, 2025) from the United Kingdom; The T1D, 2025 Diabetes Exchange Registry from the United States (De Maastricht Studie, 2025); The Maastricht Study from the Netherlands; and BIFAP, 2025, the Spanish database for Pharmaco-epidemiological Research in Primary Care	Artificial intelligence algorithms, with two different approaches: 1) Identification of patients’ subgroups characterized by specific conditions and study of treatment pathways and quality of life associated to them; and 2) Building of personalized predictive model to optimize treatment recommendations, to get evidence about risks, and to support decision making processes in HTA and clinical practice	Machine Learning model to provide personalized predictions
3 University of Bern	Relapsing-remitting Multiple Sclerosis	Dimethyl Fumarate, Glatiramer Acetate, and Natalizumab	The Natalizumab Safety and Efficacy in Relapsing Remitting Multiple Sclerosis (AFFIRM) study (Polman et al., 2006); The Comparator and an Oral Fumarate in Relapsing–Remitting Multiple Sclerosis (CONFIRM) trial (Fox et al., 2012); the Determination of the Efficacy and Safety of Oral Fumarate in Relapsing–Remitting MS (DEFINE) study (Gold et al., 2012); and the Swiss Multiple Sclerosis Cohort (The Swiss MS Cohort, 2025)	Network meta-analysis and network meta-regression methods	2 prediction models for heterogeneous effects of treatments, including i) Least Absolute Shrinkage and Selection Operator (LASSO) model and ii) pre-specified model (Chalkou et al., 2021)
4 University of York	Low to Intermediate-1 Myelodysplastic Syndrome (LR-MDS)	First-line erythropoiesis stimulating agents (ESAs)	The European Myelodysplastic Syndromes (EUMDS, 2026) registry which collected 10 years worth of data, including clinical, sociodemographic characteristics alongside treatment history and outcomes	Causal inference methods, such as target trial emulation	2 Methods: Logistic model for treatment outcomes and Beta model for EQ-5D distribution

### 2.2 Survey structure

Each case study leader filled out a survey about RWD research methods developed in their own case studies. The survey centred around a form in which the case study leaders judged whether methods in their case studies could be used by an HTA agency for a given HTA task (e.g., estimating the treatment effect, identifying the relevant comparator) in four different settings. These settings were differentiated based on whether the use i) concerned a new application or a re-evaluation, or whether the country was an “initial” (in other words early technology adopter) country with early access after market authorisation, or a “subsequent” (late technology adopter) country with delayed access (see Supplementary File 1). For each task and each setting the case study leader marked the method as one that could be used, could not be used or could potentially be used, if additional data are provided.

### 2.3 Interview structure

After the survey responses were collected and analysed, interviews were conducted with the case study leaders to gain further insights into their understanding of the applicability of their RWD research methods for HTA agencies. The interviews were semi-structured, with discussions around pre-set questions. These focused on their own RWD research methods and their applicability in given tasks and settings brought up in the survey.

## 3 Results

**FIGURE 2:**
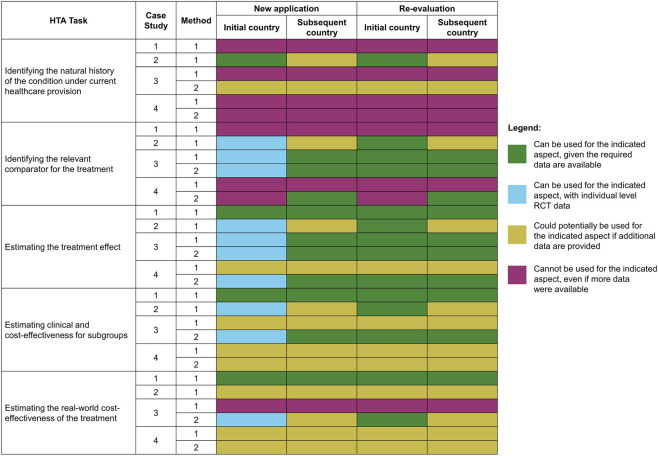
Results from case studies on the applicability of their HTA methods on aspects of HTA evaluation, where each row represents the applicability of a given method to a particular aspect. Where a case study developed more than one method (see Table 1), these are presented separately.

The analysis of the survey data revealed that the RWD research methods within the HTx case studies were geared primarily towards treatment effect aspects of HTA analysis (see Figure 2).

Note that Figure 2 includes an option of ‘Can be used for the indicated aspect with individual level RCT data’, which was not included in the original survey. During the interviews it became clear that several of the RWD research methods developed in the case studies could use RWD and RCT data with some degree of interchangeability. In some of the case studies (especially in case study 3) the research method was based on combination of data from and non-randomized studies (Hamza et al., 2023). These case studies stressed that clinical trial data could be used as a substitute for RWD in certain situations. However, it should be noted that the case studies relied on individual patient level data from randomized and single arm trials shared by the company performing the clinical study, something that is rarely available to national HTA agencies.

As can be seen in Figure 2, RWD research methods studied in HTx were primarily applicable to estimating treatment effects and estimating clinical and cost-effectiveness for subgroups, and less applicable to other tasks of HTA agencies. In terms of transferability, if they can be used for a given task, most methods allow the use of RWD from original countries both in subsequent countries and for re-evaluation. It should be noted that certain methods can be used directly for re-evaluations in the same countries, but additional data would be necessary to make it applicable in other countries. In general, several RWD research methods become more useful to HTA agencies, if additional data are provided. This is especially true for estimating clinical and cost-effectiveness for subgroups. Due to the specific time constraints for technology appraisals in the majority of countries, this indicates the necessity for HTA agencies to access this additional data in a timely manner.

In their survey responses and during the interviews the case study leaders also provided a wealth of information on other requirements for the applicability of utilise their RWD research methods in different HTA settings (see Figure 1). In terms of required data the methods vary greatly. Some methods have specific requirements, such as “outcomes every 6 months until 5 years after initial treatments with at least a hundred events recorded”. Other methods are more flexible, with increased data quantity and quality linked to decreased uncertainty of results. Generally, longer follow-up times, a more diverse patient population and larger variety in comparators were seen as advantageous, especially in terms of providing more externally valid evidence than RCT studies.

This variation in data requirements highlights the importance of flexibility in analysing RWD. Different RWD research methods could be preferable in different diseases and settings, they might also be differently suitable depending on the availability and accessibility of data. Since RWD is likely to remain heterogenous in its structure, quality and quantity, the variation of research methods is crucial to provide the necessary flexibility to utilise the best available RWD.

It became also clear from the case studies that some of the RWD research methods require highly sophisticated skills. Three groups of skills were particularly highlighted. The first is data evaluation and processing. RWD often entails ‘messy’ data from different sources with different structures or data that contains extensive abnormalities and missing values. As such, it is important that whoever is in charge of collecting the data should have extensive skills and experience in data structuring and cleaning.

A second group of skills is statistics and statistical methods. This includes theoretical skills such as epidemiology, causal inference methods and advanced Bayesian statistical modelling. It also includes practical skills with using AI and easy to access statistical software, such as the open-source R.

A third skillset that was emphasised by some of the case study leaders was epidemiological and medical knowledge, which is in some ways linked to the data skills. Since RWD in some aspects is more complicated than RCT data, it is important to understand the disease area, disease progression and treatment choices made by healthcare professionals. This knowledge would help to understand patient pathways from the data and provide guidance on how to best pre-process it.

Case study leaders indicated a few further complications for HTA agencies to utilise RWD research methods, such as cultural resistance towards RWD. Due to the limitations of RWD, a significant portion of clinicians and HTA practitioners view RWD as lower level of evidence compared to RCT data due to the facts that treatment groups were not randomly assigned or the lack of placebo comparator. As such, there is a need for education not only on the advantages of RWD, but also on the limitations of RCT data and how the RCT and RWD can complement each other.

Some of the RWD research methods are also dependent on supplementary RCT data. Combination of RWD and RCT data necessitates access to individual patient level data from RCTs, however, the sponsors of RCTs are not obliged to release such data. High-quality data about the real-world study population is needed to facilitate transfer of results from one country to another, which is not necessarily available for all countries. Others RWD research methods require data from several countries to reach the critical sample size especially in rare diseases, which necessitates the RWD to be shareable across countries.

According to the case study leaders, the most pressing issue for improving the applicability of academic RWD research methods to practical HTA usage was awareness and knowledge. HTA agencies needed increased awareness of both the advantages of using RWD and the already existing analytical tools, as well as RWD research methods in development. They also needed both the theoretical knowledge underpinning new RWD research methods and practical know-how of how to use them, in order to be able to utilize their results.

The case study leaders argued that the HTA agencies must improve collaboration with organisations that gather RWD, especially with healthcare providers. This would improve the RWD collection for HTA purposes, e.g., to ensure that RWD are more informative to address the decision problem and critical uncertainties. Improved RWD can enable HTA agencies to use RWD research methods that would otherwise be unfeasible due to data constraints.

## 4 Discussion

The findings of this study shed light on both promises and limitations newly developed RWD research methods for HTA purposes. The case studies show that RWD can be used for a range of HTA aspects, both for countries that have yet not adopted a treatment or for re-evaluation of a treatment. However, no single method is a silver bullet that is applicable for all tasks in all settings. As such, RWD analysis methods can make significant contributions to the work of HTA agencies, but as part of a wider tool set.

The current literature indicates that there is a mismatch between ambitions and abilities in the European HTA-sphere when it comes to the utilization of RWD in HTA decision making. Many European HTA agencies expressed a high willingness to incorporate RWD in their work and are working on how to do so, most often in collaboration with other HTA agencies (Strömgren and Pontén, 2020). At the same time, the literature points towards a host of challenges with using RWD, including data sources, policy structures and a lack of methods (Hogervorst et al., 2022), as well as a number of barriers to implementing new methods that utilize RWD, including “technical, regulatory, clinical, scientific and perceptional barriers” (Kamusheva et al., 2022). These challenges and barriers lead to a situation where a number of agencies do not use RWD at all, although they ideally would like to, while even the ones that do use RWD do so in a more limited capacity than they would prefer (Strömgren and Pontén, 2020).

Due to a lack of in-house resources, and with the availability of methodological competency being one of the many challenges to RWD usage, many HTA agencies report that they use collaborations and external parties to develop or conduct their RWD work (Strömgren and Pontén, 2020). Academics are one of the primary sources of external collaborators, most often used for specific tasks, such as developing or configuring an RWD research method for a specific analysis (Strömgren and Pontén, 2020). Other literature points towards the possibilities of sharing and integrating competencies on RWD between different actors in the healthcare sphere, although it also points towards significant differences in use and potential utility of RWD for different actors (Facey et al., 2020; Sola-Morales et al., 2023).

In light of the challenges and barriers with RWD usage, the agencies that do use it have done so with regards to certain limited facets of HTA (Makady et al., 2018). Often RWD is seen as a tool for especially ‘difficult cases’, where regular approaches are deemed insufficient (Hogervorst et al., 2022). Some of the literature points towards RWD being seen as especially useful in relation to highly innovative technologies (Facey et al., 2020). RWD is also markedly more common in cost-effectiveness analysis, compared to relative effectiveness analysis (Makady et al., 2018). Of note is also that, even among the agencies that utilize RWD, many of them use it reactively if an application contains RWD but are not proactively finding and using their own RWD (Strömgren and Pontén, 2020).

Apart from the challenges and barriers associated with RWD, there are also natural limitations to RWD, especially the fact that it can only be generated after the widespread use (i.e., positive reimbursement decision) of a product. As such, RWD is most useful in a follow-up capacity, such as reassessments of HTA evaluations. However, only a minority of HTA-agencies carry out reassessments on a regular basis, though among those that do so, most use RWD in these assessments (Strömgren and Pontén, 2020). The latency of RWD also makes it useful for various forms of risk-sharing arrangements (Strömgren and Pontén, 2020). For example, advanced reimbursement models, such as outcome-based or delayed payment reimbursement, might be constructed from knowledge of real-world outcomes (Ádám et al., 2022a; 2022b).

In addition, the literature points towards a range of policy factors that influence the ability to use RWD for reimbursement decisions including stable institutions, attitude and leadership, networks, competence and regulations (Strömgren and Pontén, 2020). The differences in availability of these factors have led to a situation where the policies for using RWD differ greatly between contexts and agencies (Makady et al., 2017b). This variation discourages the use of RWD, as it hampers the development of common approaches to the provision, evaluation and utility of RWD (Makady et al., 2017b). Political and economic factors make the development of frameworks for RWD usage especially problematic in Central and Eastern European countries (Kamusheva et al., 2022).

The above discussed difficulties must all be kept in mind when studying the potential for using RWD in the assessment and appraisal process by HTA agencies, especially the high degree of variation between agencies throughout Europe. However, to enable a more general discussion RWD research methods of HTx case studies, this paper has dealt with HTA agencies as a homogenous group, assuming that legal and institutional barriers allow for RWD usage. Still, there will be some exploration in terms of competencies and infrastructures needed for HTA agencies to use RWD.

The standardization of RCT has meant that it has historically been the primary, and often only, data source used by HTA agencies to support pricing and reimbursement decisions. To be able to utilize RWD, despite their diversity and irregularity, requires methods and competencies beyond what many HTA agencies have.

A basic assumption in this paper is that the RWD relating to a particular treatment is collected during the use of that technology in clinical practice. Hence, by definition, no RWD about a particular treatment can be collected prior to the first reimbursement decision. This means that at the first technology appraisal no RWD about a particular technology’s use in clinical practice is available. However, there are important HTA tasks that do not need RWD from the use of the new technologies, such as the natural disease progression or identification of relevant comparators. It could, therefore, be possible to use some RWD during the first assessment even though no data has been generated about the particular technology.

For HTA purposes it would be greatly beneficial, if an RWD research method allowed for the transfer of findings from one country to another. When a country with later access to the technology is conducting a HTA, data from use in clinical practice might have been generated in a country with earlier access. Hence, RWD from the country with earlier access might be used during the first assessment in the later access country, assuming that the results of RWD research are transferable to the setting of the evaluating country. And even more data might be available when a country conducts a re-assessment. This progression of assessment of a product in an early or late access countries is shown in Figure 1. It should be noted that transferability of each case study methodology from early access European countries to late access (mainly lower income Eastern European) countries have been discussed in more details in separate papers (Németh et al., 2023; Tachkov et al., 2024; Kaló et al., 2026).

One area in which RWD shows particular promise is within the areas of reassessment and other risk-management approaches. The existence of RWD, possibly from several countries, while additional RCT data is unlikely to exist, means that RWD could be critical in enabling these approaches. However, this is dependent on the transferability of RWD in the analytical methods and the regulatory possibilities for risk-management in HTA.

The generalisability of our conclusions may be limited, as we relied on only 4 case studies with different objectives. However, in this study it was evident that the RWD research method developers can and should increase their knowledge about how technology appraisals are performed. This would enable them to better understand how their methods can support decision making, for example, by facilitating the transferability of RWD research from one country to another. It was also evident that HTA agencies need to increase their methodological competencies in order to use the RWD that is generated. Without knowing the questions that need to be answered on one hand and on the other hand, not knowing the methods that can be used to answer these questions, data will not be used optimally.

However, reassessment and risk-management are not all that RWD might be helpful for. For countries with more limited healthcare spending possibilities, such as in Central and Eastern Europe, RWD from other countries can be a way to more accurately assess the effectiveness and cost-effectiveness of a new technology. This way, these countries can leverage their often-delayed access into better HTA analysis. Or, if countries with higher healthcare spending possibilities start to demand RWD in their initial HTA analysis, especially if companies continue to be reluctant in supplying individual level RCT data, other countries can negotiate earlier access and better prices for a healthcare technology in return for enabling the companies to gather the required RWD.

## Data Availability

The data that support the findings of this study are available in aggregated form in the article/Supplementary Material. Further inquiries can be directed to the corresponding author, though access may be restricted due to privacy concerns.
