# Supplementary material for: Applicability of academic real-world data research in the case studies of the HTx project to practical health technology assessment work
Source: Front Pharmacol. 2026 Mar 13;17:1650552. doi: 10.3389/fphar.2026.1650552 (PMC13021581; doi:10.3389/fphar.2026.1650552)
Supplement: Supplementary file 1 [file Supplementaryfile1.docx]

# Appendix I.

# HTA-Compatibility Review

Case Study:

Name of Method:

(Please fill out one form for each method in your CS)

In the spring of 2024, TLV will conduct a study to follow-up on the results of the HTx project. It aims to evaluate the usability of the scientific results from the work packages in terms of their applicability in the practical work of HTA-agencies. To do this, we need your input and so we hope that you’ll be willing to fill out the following form and participate in upcoming interviews.

Below is a table that we would want to invite you to fill out, as well as a number of questions we would like you to answer. They all relate to the evaluation of an HTA-application for a specific pharmaceutical or health care technology to a given HTA-agency.^[[1]](#footnote-2)^

A basic assumption is that results can, under the right circumstances, be translated from one country to another country. This is what we define as transferability. There might be factors such as demographic factors, disease burden or health care systems that differ between countries but by taking these factors into account, it should be possible to transfer results from one setting into another to some extent. This means that the results of a study that is performed following introduction of a treatment in a country with early access (named “initial country” below) can be used to inform HTA-evaluations in a country that has later access (subsequent country) to the treatment.

In this exercise we assume that a new treatment is introduced in the same therapeutic area as your case study. The developer of that treatment seeks reimbursement by an HTA organization, and a cost-effectiveness model is used for evaluation of the treatment. The methodology developed/used in your case study is to be used in this evaluation. However, different methods can be used for different purposes in different stages of the life cycle of HTA. In this exercise we want to evaluate when and how your methodology can be used.

In the form, we first want you to indicate for which aspects of the HTA evaluation your method could be useful. Please indicate in the below matrix if your method:

- **can** be used for each aspect, given the required data are available (“yes”);
- **cannot** be used for the indicated aspect, even if more data were available (“no”), or;
- **could potentially** be used if additional data are provided (“with additional information”).^[[2]](#footnote-3)^

|  | New application in initial country | New application in subsequent country | Re-evaluation in initial country | Re-evaluation in subsequent country |
| --- | --- | --- | --- | --- |
| Identifying natural history of the condition under current health care provision |  |  |  |  |
| Identifying the relevant comparator for the treatment seeking reimbursement |  |  |  |  |
| Estimating the treatment effect of the treatment seeking reimbursement |  |  |  |  |
| Estimating subgroup-optimised clinical/cost effectiveness |  |  |  |  |
| Estimate the real-world cost-effectiveness of the treatment applying for reimbursement |  |  |  |  |

In what quantity and for how long would real world data need to be collected in order to be useful for your method?

Describe the skills required to use your method in HTA-evaluations:

Describe other potential limitations that can prevent your method from being used for HTA-evaluations, such as rare or expensive soft- or hardware:

How could your method be adapted to increase its applicability to the various stages of HTA-evaluation?

**Appendix I. Explanation of terms**

Initial country: A country that introduces a HT-product before sufficient real-world data has been collected in order to use RWD in your model.

Subsequent country: A country that introduces a HT-product after sufficient real-world data has been collected in order to use RWD in your model.

Additional data: Can be information about population demographics or time course/burden of disease.

1. Referred to as the application/applicant in the matrix below. [↑](#footnote-ref-2)
2. For more information on the matrix, see appendix I. [↑](#footnote-ref-3)
